# Vasohibin-1 Expression Can Predict Pathological Complete Remission of Advanced Bladder Cancer with Neoadjuvant Chemotherapy

**DOI:** 10.1245/s10434-024-15009-1

**Published:** 2024-02-20

**Authors:** Minami Omura, Takeo Kosaka, Hiroaki Kobayashi, Keisuke Shigeta, Kazuhiro Matsumoto, Satoshi Hara, Eiji Kikuchi, Shuji Mikami, Hideyuki Saya, Yasufumi Sato, Mototsugu Oya

**Affiliations:** 1 Department of Urology, Keio University School of Medicine, Tokyo, Japan; 2 Department of Urology, Kawasaki Municipal Hospital, Kanagawa, Japan; 3 Department of Urology, St. Marianna University School of Medicine, Kanagawa, Japan; 4 Department of Pathology, Keio University School of Medicine, Tokyo, Japan; 5 Division of Gene Regulation, Institute for Advanced Medical Research, Graduate School of Medicine, Keio University, Tokyo, Japan; 6 Department of Vascular Biology, Institute of Development, Aging and Cancer, Tohoku University, Miyagi, Japan

**Keywords:** Angiogenesis, Biomarkers, Bladder cancer, Chemotherapy, Pathological outcome

## Abstract

**Background and purpose:**

Neoadjuvant chemotherapy (NAC) is a well-established standard practice in invasive bladder cancer (BCa), however patient selection remains challenging. High expression of vasohibin-1 (VASH1), an endogenous regulator of angiogenesis, has been reported in high-grade and advanced BCa; however, its prognostic value for chemotherapy outcomes remains unexplored. In this study, we sought to identify biomarkers of chemotherapy response focusing on the relationship between angiogenesis and tissue hypoxia.

**Methods:**

Forty Japanese patients with BCa who underwent NAC and radical cystectomy were included in the present analysis. We compared the immunohistochemical expression of CD34, VASH1, and carbonic anhydrase 9 (CA9) between patients who achieved tumor clearance at operation (ypT0) and those with residual disease after cystectomy.

**Results:**

There were 19 patients in the ypT0 group, while the remaining 21 patients had residual tumors at operation. Patients in the ypT0 group had high microvessel density (*p =* 0.031), high VASH1 density (*p* < 0.001), and stronger CA9 staining (*p* = 0.046) than their counterparts. Multivariate analysis identified microvessel and VASH1 density as independent predictive factors for pathological ypT0 disease (*p* = 0.043 and 0.002, respectively). The 5-year recurrence-free survival rate was higher in the high VASH1 density group than in the low VASH1 density group (66.3% vs. 33.3%, *p* = 0.036).

**Conclusion:**

VASH1 density is a potential therapeutic biomarker for chemotherapy response in BCa.

**Supplementary Information:**

The online version contains supplementary material available at 10.1245/s10434-024-15009-1.

Bladder cancer (BCa) is the 11th most frequent cancer worldwide.^1^ Patients with muscle-invasive bladder cancer (MIBC) are known to have an aggressive clinical course,^2^ and neoadjuvant chemotherapy (NAC) is currently one of the standard treatment strategies.^3^ However, patients with a poor response to NAC are bound to experience both delays in operation and treatment-related toxicity with poor outcomes.^4^ Thus, biomarkers for identifying patients who are likely to respond to classical NAC are needed.

Angiogenesis is one of the essential factors in tumor progression and is often promoted in solid tumors to increase oxygen and nutrient supply. Ironically, excessive angiogenesis creates abnormal vasculature with high permeability,^5–7^ often resulting in tissue hypoxia.^8^ Cellular hypoxia then acts as a trigger for accelerating angiogenesis, serving as both an outcome and a stimulus of angiogenesis. Therefore, angiogenesis and hypoxia are inseparable.

CD34 staining of epithelial cells and calculation of microvessel density (MVD) serve as biomarkers of angiogenesis.^9^ MVD quantifies vasculature but fails to distinguish original vessels from neovascularized vessels, and is insufficient for evaluating ongoing angiogenic activity. As a marker of ongoing angiogenic activity, we focused on vasohibin-1 (VASH1), an endogenous regulator of angiogenesis that is specifically expressed in activated endothelial cells (ECs).^10^ Proangiogenic factors, such as vascular endothelial growth factor, increase the synthesis of VASH1 over 24 hours,^11^ and VASH1 acts as a negative feedback regulator of angiogenesis in the termination zones, which is the most distal area of mature vasculature, characterized by mural cell lineage and non-proliferative ECs.^12^ High VASH1 expression has been reported in high-grade and advanced BCa^13^; however, the prognostic value of VASH1 in relation to chemotherapy outcomes remains unclarified.

In the present study, we sought to identify potential biomarkers of chemotherapy response. To this end, we evaluated the expression and relationship between MVD and VASH1 in transurethral resection (TUR) specimens acquired before NAC, focusing on angiogenic activity in the tumor microenvironment.

## Methods

All procedures performed in this study involving human participants were in accordance with the ethical standards of the institutional and national research committee and with the 1964 Helsinki Declaration and its later amendments or comparable ethical standards. The study was approved by the Institutional Review Board of Keio University under No. 2013-0095, and that of the Kawasaki Municipal Hospital under 2018-33.

Informed consent was obtained from all individual participants included in the study.

### Patients

Between 18 December 2008 and 18 June 2020, we reviewed 226 Japanese patients with BCa who were diagnosed with cTa-T4NXM0 and treated by open radical cystectomy (RC) at Keio University Hospital and Kawasaki Municipal Hospital. A total of 172 patients without NAC, 3 patients receiving radiation therapy, and 11 patients with missing data were excluded, and 40 patients receiving RC and NAC were included in the present analysis. Chemotherapy regimens included gemcitabine and cisplatin or methotrexate, vinblastine, adriamycin, and cisplatin.

### Surgical Management and Pathological Evaluation of Advanced Urothelial Carcinoma

Patients included underwent standard open RC with urinary diversion reconstruction with an ileal conduit, neobladder, or cutaneous ureterostomy. Regional lymphadenectomy was performed, including the bilateral external iliac, internal iliac, and obturator lymph nodes. After surgery, patients were generally followed up at least every 3–4 months for 2 years, then every 6 months until 5 years, and annually thereafter. Physical examination and routine blood biochemical tests were performed in each follow-up visit. Diagnostic imaging, consisting of computed tomography of the chest/abdomen/pelvis was performed every 6 months for 5 years and then annually or when clinically indicated.

Tumors were staged according to the 2017 American Joint Committee on Cancer/Union for International Cancer Control TNM classification, and graded based on the 2016 World Health Organization classification.^14^

### Immunohistochemistry

The tumor specimens for immunohistochemical staining were obtained from the last TUR procedure before NAC. Sections were deparaffinized in xylene and rehydrated using a descending series of ethanol. After antigen retrieval at pH = 9, the endogenous peroxidase activity was blocked using 0.3% hydrogen peroxidase. Tissue sections were then incubated with a blocking solution containing 6% dry milk in phosphate-buffered saline (PBS). The primary antibodies used were mouse monoclonal antibodies (mAbs): anti-human VASH1 mAb diluted at a concentration of 4 µg/mL, anti-CD34 (Nichirei Biosciences, Tokyo, Japan) mAb diluted at a concentration of 250 µg/mL, and anti-carbonic anhydrase 9 (CA9; Nichirei Biosciences) mAb diluted at a concentration of 5 µg/mL.

Tissue sections were then incubated with secondary antibodies (Histofine Simple Stain MAX PO (M); Nichirei Biosciences) after washing with PBS. The color was developed using 3,3-diaminobenzidine tetrahydrochloride in 50 mM Tris-HCl (pH = 7.5) containing 0.005% hydrogen peroxide. Finally, the sections were counterstained with hematoxylin.

Appropriate negative control slides were prepared by substituting the primary antibody with the immunoglobulin fraction of non-immune mouse serum at the same concentration in each staining run.

### Assessment of Immunohistochemistry Staining Results

Two authors (MO and HK) evaluated immunoreactivity by counting the microvessels near the tumor using an Olympus IX71 microscope (Olympus, Tokyo, Japan). The authors were blinded to the clinical course of the patients, and the average number counted by the two investigators was used for subsequent analyses. The microvessels were identified based on their structure; specifically, the lumen was lined with ECs positively stained for CD34. Any immunostained EC or cluster separated from the adjacent vessels was counted as a single microvessel, even in the absence of the vessel lumen. After scanning the tissue sections, areas with a high concentration of vessels were selected and counted at high magnification (×200). The highest number of microvessels in the hotspot was used to determine the MVD. The cut-off value for MVD, derived from the median value, was set at 65 vessels/mm^2^.

VASH1-positive signals were counted in the hotspot area, where the highest number of vessels positive for anti-CD34 was found. We defined the number of VASH1-positive vessels/mm^2^ as VASH1 density. High VASH1 density, derived from the median value, was defined as > 30 vessels/mm^2^.

**Fig. 1:**
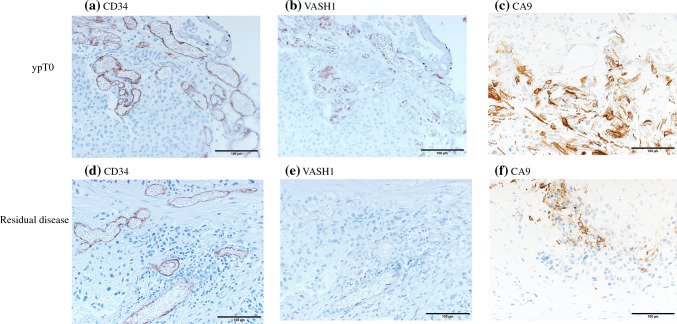
Immunohistochemical staining of **a** CD34, **b** VASH1, and **c** CA9 in tissues from patients with ypT0 BCa, showing predominantly stronger staining than that of **d** CD34, **e** VASH1, and **f** CA9 in tissues from patients with ypT4a BCa (magnification: ×200). *VASH1* vasohibin-1, *CA9* carbonic anhydrase 9, *BCa* bladder cancer

CA9 expression was evaluated as the percentage of the entire tumor sample that stained positive for CA9. Tumor staining in > 35% of cells was considered wide expression, following a previous report by Klatte et al.^15^. Staining intensity was evaluated on a scale of 0–3 (0: negative, 3: strong) [electronic supplementary material (ESM) Fig. 1].

### Statistical Analysis

Quantitative data are expressed as the median and standard deviation. Factors were dichotomized and compared using the Chi-square test, and logistic regression was used to assess the significance of differences between proportions.

Recurrence-free survival (RFS) was calculated as the duration from RC to the date of disease recurrence (local recurrence including urinary tract recurrence, such as upper urinary tract and/or urethral recurrence) and/or distant metastasis. Cancer-specific survival (CSS) was defined as the period from RC to cancer-related death due to UC. RFS and CSS rates were estimated using the Kaplan–Meier method and analyzed using the log-rank test. Multivariate analysis of disease recurrence and cancer-specific death was performed using a Cox proportional hazards model with stepwise forward selection.

In all statistical analyses, the tests were two-sided and statistical significance was set at *p* < 0.05. All analyses were performed using the SPSS version 27.0 statistical software package (IBM Corporation, Armonk, NY, USA).

## Results

Among the 40 patients who received NAC followed by RC, 19 had complete tumor elimination at operation (ypT0), while the remaining 21 had residual tumors at operation. Of the 40 patients, 34 were male and 6 were female, and the median age was 68 years (range 43–81). The remaining clinicopathological features of the patients are presented in Table 1.

**Table 1:** Clinical and pathological characteristics of NAC + RC patients

Patient characteristics	Total [*n* = 40 (%)]
Age at diagnosis, years	68.0 ± 9.184
Sex	
Male	34 (85.0)
Female	6 (15.0)
Clinical T stage	
cT1	1 (2.5)
cT2	21 (52.5)
cT3	15 (37.5)
cT4	3 (7.5)
Clinical N stage	
cN0	33 (82.5)
cN1	3 (7.5)
cN2	4 (10.0)
MVD, /mm^2^	
< 65	18 (45.0)
≥ 65	22 (55.0)
VASH1 density, /mm^2^	
< 30	21 (52.5)
≥ 30	19 (47.5)
CA9 positivity in tumor cells, %	
< 35	29 (72.5)
≥ 35	11 (27.5)
CA9 intensity	
< 2	13 (32.5)
≥ 2	27 (67.5)

*CA9* carbonic anhydrase 9, *MVD* microvessel density, *NAC* neoadjuvant chemotherapy, *RC* radical cystectomy, *VASH1* vasohibin-1

The univariate analysis results are presented in Table 2 and representative staining results are shown in Fig. 1. The mean MVD was 86.3 vessels/mm^2^ in the whole cohort. The mean MVD was 114.8 vessels/mm^2^ and 60.4 vessels/mm^2^ in the ypT0 group and control group of patients with residual tumor in the post-cystectomy specimen, respectively. When dichotomized, high MVD was observed in 14/19 ypT0 patients and 8/21 control patients, and the difference between the two groups was statistically significant (73.7% vs. 38.1%, *p* = 0.031). The mean VASH1 density was 35.1 vessels/mm^2^ in the whole cohort, 55.8 vessels/mm^2^ in the ypT0 group, and 16.4 vessels/mm^2^ in the control group. High VASH1 density was observed in 15/19 ypT0 patients and 4/21 control patients; ypT0 patients displayed significantly higher rates of high VASH1 population than their counterparts (78.9% vs. 19.0%; *p* < 0.001).

**Table 2:** Univariate and multivariate analysis for factors related to ypT0 pathology in NAC + RC patients

Patient characteristics	ypT0 [*n* = 19 (%)]	More than ypT0 [*n* = 21 (%)]	Univariate	Multivariate	
			*p*-Value	*p*-Value	OR
Age at diagnosis, years					
< 70	7 (36.8)	11 (52.4)	0.360		
≥ 70	12 (63.2)	10 (47.6)			
Sex					
Male	17 (89.5)	17 (81.0)	0.664		
Female	2 (10.5)	4 (19.0)			
Clinical T stage					
< T3	3 (15.8)	18 (4.8)	0.067		
≥ T3	16 (84.2)	3 (95.2)			
Clinical N stage					
cN0	14 (73.7)	19 (90.5)	0.226		
≥ cN1	5 (26.3)	2 (9.5)			
Histology					
Pure UC	12 (63.2)	17 (81.0)	0.293		
Histological variant	7 (36.8)	4 (19.0)			
MVD, /mm^2^			0.031	0.043	
< 65	5 (26.3)	13 (61.9)			6.803
≥ 65	14 (73.7)	8 (38.1)			1
VASH1 density, /mm^2^			< 0.001	0.002	
< 30	4 (21.1)	17 (81.0)			17.544
≥ 30	15 (78.9)	4 (19.0)			1
CA9 positivity in tumor cells, %			1		
< 35	14 (73.7)	15 (71.4)			
≥ 35	5 (26.3)	6 (28.6)			
CA9 intensity			0.046	0.102	
< 2	3 (15.8)	10 (47.6)			
≥ 2	16 (84.2)	11 (52.4)			

*CA9* carbonic anhydrase 9, *MVD* microvessel density, *NAC* neoadjuvant chemotherapy, *OR* odds ratio, *RC* radical cystectomy, *UC* urothelial carcinoma, *VASH1* vasohibin-1

Additionally, we evaluated tissue hypoxia using CA9, a member of the carbonic anhydrase family known for its role as a marker of tissue hypoxia. The mean CA9 positivity in tumor cells in our cohort was 21.4% in the ypT0 group and 21.9% in the control group, with no significant difference between the two groups. The mean CA9 staining intensity was 2.0 in the whole cohort, 2.6 in the ypT0 group, and 1.8 in the control group. High-grade CA9 staining was observed in 16/19 ypT0 patients and 11/21 control patients, with a significant difference between the two groups (84.2% vs. 52.4%, *p* = 0.046). There was no correlation between CA9 and the angiogenic markers (ESM Fig. 2). Multivariate analysis showed that MVD and VASH1 density were independent predictive factors for pathological ypT0 disease (*p* = 0.043 and 0.002, respectively) [Table 2].

**Fig. 2:**
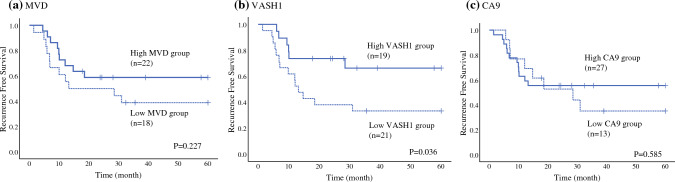
Kaplan–Meier survival curves of RFS between the **a** high and low MVD groups, **b** high and low VASH1 density groups, and **c** strong and weak CA9 staining groups in BCa treated with NAC + RC (*n* = 40). *RFS* recurrence-free survival, *MVD* microvessel density, *VASH1* vasohibin-1, *CA9* carbonic anhydrase 9, *BCa* bladder cancer, *NAC* neoadjuvant chemotherapy, *RC* radical cystectomy

We evaluated the 5-year RFS and CSS rates among the patient groups. Regarding RFS, no difference in prognosis was observed between the groups stratified by MVD and CA9, whereas in the VASH1 group, the 5-year RFS rates were higher in the high VASH1 density group, as shown in Fig. 2 (5-year RFS: low vs. high, 33.3% vs. 66.3%; *p* = 0.036). No significant differences were observed in CSS among the three groups (ESM Fig. 3).

## Discussion

In this study, we evaluated intertumoral MVD, VASH1 density, and CA9 staining intensity in 40 patients with BCa receiving NAC, to evaluate their predictive value for chemotherapy response. In the ypT0 cohort, high MVD, high VASH1 density, and strong CA9 staining were observed more frequently in patients who achieved tumor clearance at the time of the operation. Multivariate analysis showed that MVD and VASH1 remained independent predictive factors for tumor elimination. Patients with high VASH1 density also had better RFS than their counterparts. To the best of our knowledge, this is the first study to report VASH1 density as a biomarker for NAC outcomes in patients with BCa.

During tumor formation and expansion, tissue hypoxia triggers a series of responses, including angiogenesis.^16^ Accelerated angiogenesis eventually leads to the formation of irregular vasculature with structural abnormalities and hyperpermeability,^17^ resulting in increased interstitial fluid pressure and low delivery of chemotherapeutic drugs.^18–20^ In these cells, extracellular accumulation of cytotoxic drugs has been reported and may also direct chemotherapy resistance.^21^ We therefore hypothesized that the increased expression of angiogenesis and tumor hypoxia markers may be predictive of chemotherapy resistance. We selected CD34 and VASH1 as markers of angiogenesis, and CA9 as a marker of hypoxia.

MVD has been associated with disease progression, recurrence, and poor survival outcomes in both non-muscle-invasive bladder cancer and MIBC.^9,22^ However, due to the time period of past studies, NAC rates were low and evaluation in patients after NAC has been undefined. In our cohort, patients in the ypT0 group tended to have a higher MVD than their counterparts, supporting our hypothesis that under NAC treatment, the degree of angiogenesis may predict treatment sensitivity. However, MVD does not necessarily reflect ongoing activity and is insufficient for evaluating the quality of newly formed blood vessels.

Overpromotion of angiogenesis leads to abnormal, hyperpermeable vasculature, and apart from vessel density, maturation and structural stabilization of vessels have been defined by lineage of pericytes and vascular smooth muscle cells.^23^ VASH1 is known to act as a negative feedback factor at the terminal zones of ongoing angiogenesis.^11^ In an in vivo study using a melanoma xenograft model, VASH1 overexpression led to reduction of large vessels and increase in vessels staining positive for mural cell markers, supporting maturation of vessels.^24^ VASH1 is a potent marker that compensates for the forementioned weak points of MVD.

VASH1 in urothelial carcinoma has been previously reported in only two studies.^13,25^ Zhang et al. investigated VASH1 density in 50 all-stage patients with BCa (17 with MIBC) and reported worse 5-year OS and progression-free survival in patients with high VASH1 density (*p* < 0.01).^13^ We previously reported VASH1 expression in 117 patients with upper tract urothelial carcinoma and observed worse 5-year RFS and CSS in patients with high VASH1 density.^25^ In these reports, most of the patients did not receive NAC.

In our study, TUR specimens from ypT0 patients showed predominantly stronger VASH1 staining than those from patients with residual disease at surgery. VASH1 may have dual roles as a predictor of patient outcomes. In early-stage patients and NAC-free patients, VASH1 expression may serve as a negative predictive factor for survival, whereas in patients with advanced disease and NAC, VASH1 may serve as a positive predictor of chemotherapy response and possibly outcome. This is understandable, for if VASH1 is related to tumor vascular quality, in natural disease course, high VASH1 tumors are likely to have a stable vascular structure, enabling growth and circulation of tumor cells. At the same time, this vessel structure enhances delivery of cytotoxic drugs with elevated treatment response. Thus, patients with high VASH1 may be most likely to benefit from NAC.

As another approach to evaluate vessel quality and the difference in the tumor microenvironment in tumors with angiogenesis promotion, we focused on tumor hypoxia. CA9 has been known as a representative marker of hypoxia and has been studied in UC. Todenhöfer et al. explored CA9 mRNA expression in patients with MIBC treated with NAC and reported no significant association with pathological outcome.^26^ Hoskin et al. reported that OS negatively correlates with CA9 positivity in tumor cells (negative vs. low vs. high; *p* = 0.021).^27^ However, the rates of NAC were low in these studies and, to our knowledge, no previous study on the chemotherapy response based on CA9 tissue immunohistochemical staining has been reported in urothelial cancer.

In our study, no significant difference was detected among the groups divided by CA9 positivity in tumor cells, although the staining intensity was stronger in the ypT0 group. One reason for this may be the variation in the tumor in the TUR specimens. After multivariate analysis, the CA9 staining intensity was not defined as an independent predictor of chemotherapy response. CA9 and hypoxia is a factor that changes dynamically during angiogenic activity and is difficult to quantify without chronical factors. Thus, we concluded that although connection between angiogenesis and hypoxia is strong, as a biomarker for chemotherapy response, VASH1 is the most potent and fit.

With this finding, future hopes are high. Currently, with the rise of next-generation sequencing, genomic analysis has been performed in various solid tumors with the aim of discovering mechanisms of carcinogenesis, adoption, and therapy response. Genomic analysis with increased numbers of patients may lead to the discovery of underlying genomic alterations in patients with a relation to vasohibin expression, which may explain the reasons of heterogeneity in chemotherapy response in invasive BCa patients. Additionally, VASH1 is a measurable marker in blood serum concentration levels, which is easily obtained and quantified.^28^ If the relation between VASH1 and chemotherapy response becomes established, it could be a convenient marker for stratification and selection of patients who are candidates for NAC. Furthermore, changes in practice based on VASH1 level may also become possible. Recently, in early-stage breast cancer, where NAC and surgical resection is standard treatment, a phase II prospective trial of surgery omission in patients with pCR in vacuum-assisted core biopsy after initial chemotherapy have been reported, with promising results.^29^ Similarly, if we could select patients with high VASH1 density to undergo a TUR after initial chemotherapy and confirm that they are pCR, studies of bladder conservation therapy may also be possible.

The present study has several limitations. This study was performed retrospectively with a limited number of patients. Our cohort, consisting of patients with MIBC undergoing NAC and RC, was high-stage and was expected to have a poor prognosis compared with other patient populations. Thus, it is difficult to compare the results with those of previous studies focusing on survival outcomes. In addition, the specimens were regularly collected after resection was completed, and the variation in time to fixation may have affected hypoxia in individual samples.

## Conclusion

TUR specimens in patients achieving ypT0 after NAC showed predominantly stronger MVD, VASH1, and CA9 staining than those with residual disease at surgery. Higher MVD and VASH1 density were independent predictors of chemotherapy response in patients receiving NAC and RC. High VASH1 density was also predictive of a better 5-year RFS. Thus, VASH1 density may be a potential therapeutic biomarker for chemotherapy response in BCa.

### Supplementary Information

Below is the link to the electronic supplementary material.

**Supplementary Fig. 1** Visualized scale of CA9 staining.* CA9* carbonic anhydrase 9

**Supplementary Fig. 2** Correlation between MVD, VASH1, and CA9 expression,* MVD* microvessel density,* VASH1* vasohibin-1,* CA9* carbonic anhydrase 9

**Supplementary Fig. 3** Kaplan–Meier survival curves of CSS between the (**a**) high and low MVD groups, (**b**) high and low VASH1 density groups, and (**c**) strong and weak CA9 staining group in BCa treated with NAC + RC (*n* = 40).* CSS* cancer-specific survival,* MVD* microvessel density,* VASH1* vasohibin-1,* CA9* carbonic anhydrase 9,* BCa* bladder cancer,* NAC* neoadjuvant chemotherapy,* RC* radical cystectomy
