# Supplementary figures and images for: Vasohibin-1 Expression Can Predict Pathological Complete Remission of Advanced Bladder Cancer with Neoadjuvant Chemotherapy
Source: Ann Surg Oncol. 2024 Feb 20;31(5):2951–8. doi: 10.1245/s10434-024-15009-1 (PMC10997694; doi:10.1245/s10434-024-15009-1)

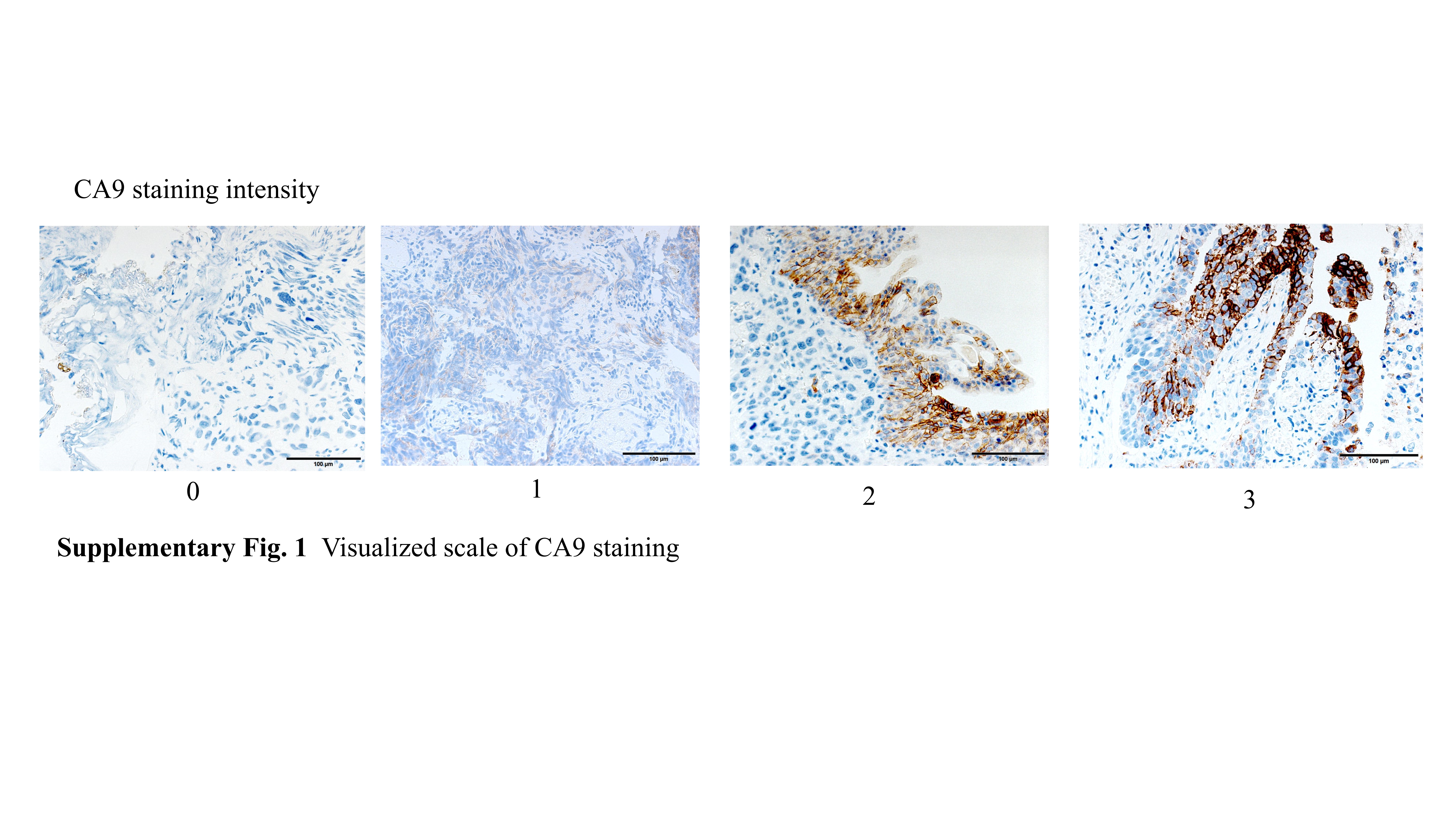

Supplement: Supplementary file 1 — Supplementary Fig. 1 Visualized scale of CA9 staining. CA9 carbonic anhydrase 9 [file 10434_2024_15009_MOESM1_ESM.jpg]

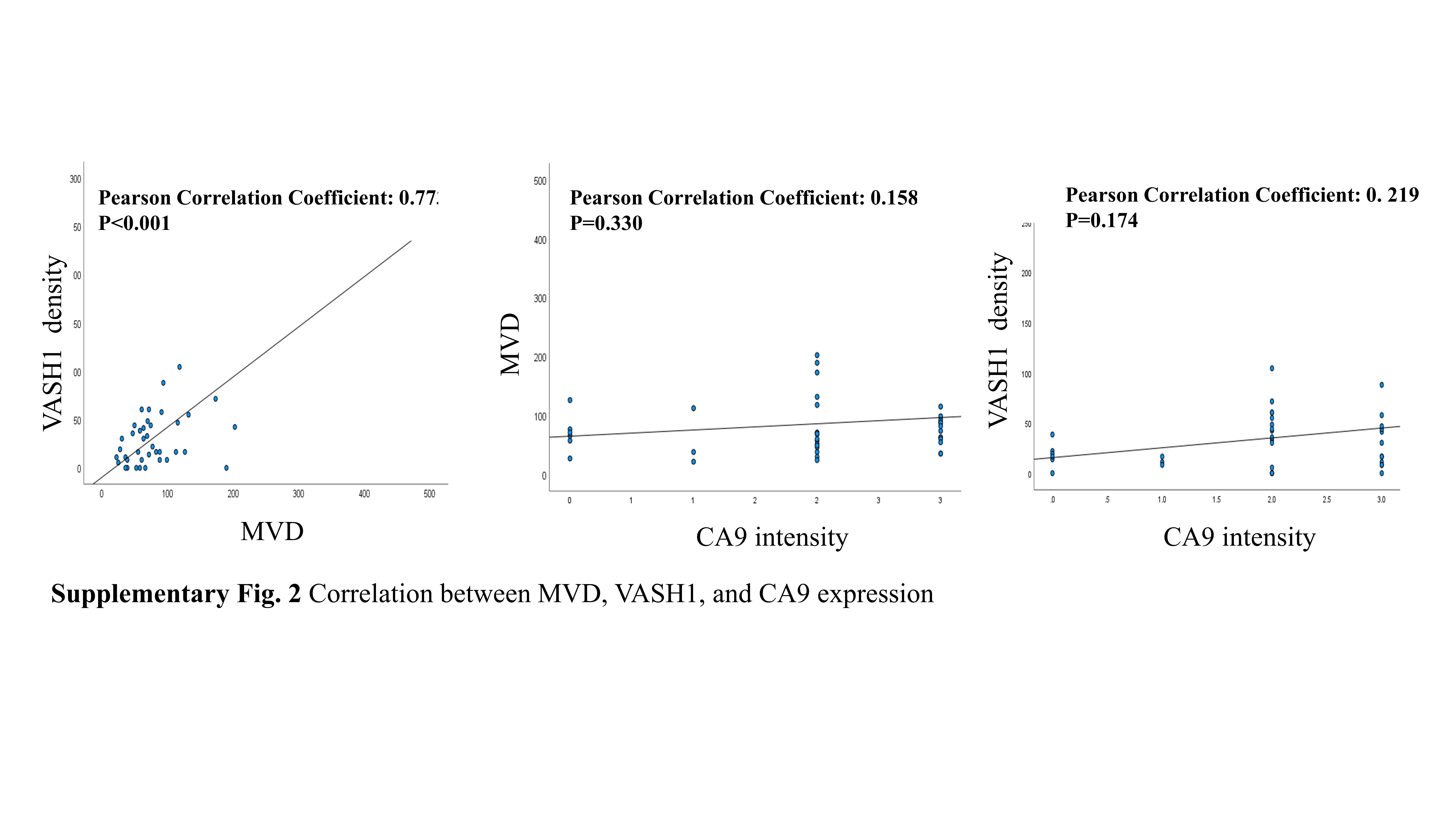

Supplement: Supplementary file 2 — Supplementary Fig. 2 Correlation between MVD, VASH1, and CA9 expression, MVD microvessel density, VASH1 vasohibin-1, CA9 carbonic anhydrase 9 [file 10434_2024_15009_MOESM2_ESM.jpg]

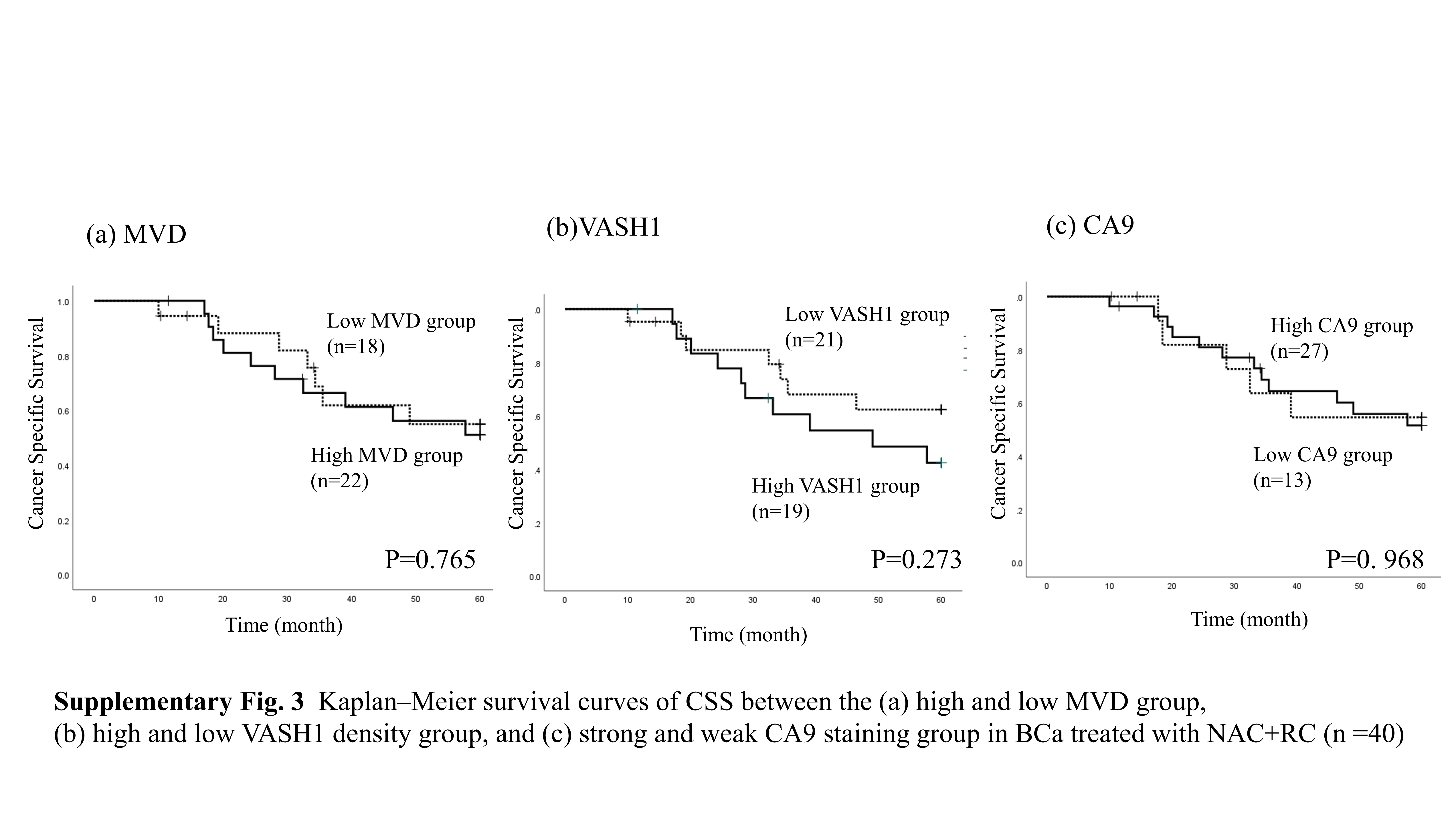

Supplement: Supplementary file 3 — Supplementary Fig. 3 Kaplan–Meier survival curves of CSS between the (a) high and low MVD groups, (b) high and low VASH1 density groups, and (c) strong and weak CA9 staining group in BCa treated with NAC + RC (n = 40). CSS cancer-specific survival, MVD microvessel density, VASH1 vasohibin-1, CA9 carbonic anhydrase 9, BCa bladder cancer, NAC neoadjuvant chemotherapy, RC radical cystectomy [file 10434_2024_15009_MOESM3_ESM.jpg]
